# The influence of goutweed (Aegopodium podagraria L.) tincture and metformin on the carbohydrate and lipid metabolism in dexamethasone-treated rats

**DOI:** 10.1186/s12906-016-1221-y

**Published:** 2016-07-22

**Authors:** O. V. Tovchiga

**Affiliations:** Department of Pharmacology and Drug Toxicology, National University of Pharmacy, Pushkinskaya Str. 53, Kharkiv, 61002 Ukraine

**Keywords:** *Aegopodium podagraria L*, Goutweed, Dexamethasone, Metformin, Rats, Combined drugs

## Abstract

**Background:**

Diabetes mellitus and metabolic syndrome are the common problems of the modern society. The interest in herbal medicines increases, and often they are used in combination with conventional drugs. *Aegopodium podagraria L.* (goutweed) is a plant widely used in traditional medicine. Hypoglycemic effect of goutweed aerial part tincture has been previously shown in alloxan-induced diabetic mice and in rats receiving excess of fructose and hydrochlorothiazide. The effects of co-administration of the tincture with widely used antihyperglycemic drugs have not been verified. The objective of this study is to determine the efficacy of goutweed tincture and its combination with metformin using the model reproducing the pathogenetic mechanisms of the metabolic syndrome and type 2 diabetes.

**Methods:**

The animals were divided into 5 groups, as follows: intact control, dexamethasone (untreated), dexamethasone + metformin, 50 mg/kg; dexamethasone + *A. podagraria* tincture, 1 ml/kg intragastrically; dexamethasone + metformin, 50 mg/kg intragastrically + *A. podagraria* tincture, 1 ml/kg intragastrically. Dexamethasone was used at a dose of 5 mg/kg subcutaneously for 5 days. Insulin tolerance test and oral glucose tolerance test were performed, triglycerides, total lipids, total and HDL cholesterol content in plasma were determined, LDL cholesterol content was calculated, glycogen content in the liver was measured.

**Results:**

Goutweed tincture combined with metformin increased its effect on the basal glycemia and on the results of the short insulin test. In the oral glucose tolerance test the lowest area under glucose curve and average glycemia value were seen in animals receiving this combination. Only metformin tended toward the reduction of liver glycogen. The decrease in triglycerides and increment of HDL cholesterol content (caused by the tincture), as well as tendency towards the decrease in total lipids level (caused by metformin) were observed against a background of the investigated combination, though the ability of GW tincture to reduce LDL cholesterol content and the same tendency seen against a background of metformin were eliminated when these preparations were administered together.

**Conclusion:**

It has been shown that goutweed tincture combined with the respectively low dose of metformin partially increases the efficacy of the latter in dexamethasone-treated rats.

**Graphical abstract:**

Goutweed tincture combined with the respectively low dose of metformin partially increases the efficacy of the latter in dexamethasone-treated rats
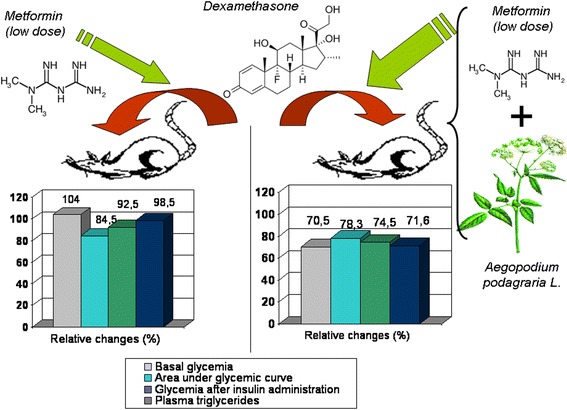

## Background

In recent years much attention has been paid to the improvement of the therapy of metabolic syndrome and type 2 diabetes. The total number of people with diabetes is projected to rise from 171 million in 2000 to 366 million in 2030 [1]. Among the drugs that provide control of glycemia and reduce cardiovascular risk factors metformin is widely accepted first line agent with additional benefits such as improvements in endothelial dysfunction, hemostasis and oxidative stress, insulin resistance, lipid profiles, and fat redistribution [2]. At the same time, patients are becoming more interested in traditional herbal medicines. Herbal drugs are frequently used with conventional drugs as complementary agents, often without medical advice [3]. Still the safety of the herbal drugs is often overestimated. Medicinal plants contain a plethora of active compounds that is a prerequisite of an increased risk of potential herb-drug pharmacokinetic and pharmacodynamic interactions, which, in case of diabetes, are highly possible considering lifelong treatment [4]. On the other hand, herbal drugs may stipulate favourable pharmacological effects and enhance the efficacy of known antihyperglycemic drugs. A great body of evidence exists about antihyperglycemic and related effects of herbal medicines [5, 6]. The preclinical studies of the efficacy and safety of herbal drugs combinations with antidiabetic medicines have intensified recently. Several herbal preparations and plant constituents increase efficacy of metformin on the model of alloxan-induced diabetes [7], streptozotocine-induced diabetes [8], in vitro and ex vivo [9].

Our efforts are focused on the verification of pharmacological activity of the drugs obtained from the aerial part of *Aegopodium podagraria L.* (goutweed). It is a perennial plant of the Apiaceae family indigenous to Europe, Siberia, the Caucasus, Kazakhstan and Central Asia mountainous regions and has been naturalized in North America and Australia. The plant is ubiquitous, widely used in traditional medicine, and consumed as vegetable. The low toxicity level of goutweed preparations has been confirmed experimentally [10]. Hydroxycinnamic acids, flavonoids, coumarins, polyacetylene compounds, essential oil components, micro- and macroelements were identified in *A. podagraria* aerial part. The capillary electrophoresis method was applied to establishing electrophoretic fingerprints of leaves and stems of *A. podagraria* [11, 12].

Goutweed aerial part tincture and dry extract exert protective effects in alloxan-induced diabetic mice [13], the tincture renders hypoglycemic effect under the conditions of metabolic disorders induced by fructose and hydrochlorothiazide in rats [14], it also may cause hypoglycemic action in intact animals [15]. *A. podagraria* preparations through different mechanisms counteract hyperuricemia [10, 11] that is an important link in the pathogenesis of metabolic syndrome [16]. It is well known that kidney and liver are among the organs that undergo pathological changes in diabetes, and the additional benefits of goutweed preparations are nephroprotective and hepatoprotective action proven in several experimental models [10, 11, 13]. So, pharmacological properties of *A. podagraria* can be of great value in metabolic syndrome and diabetes type 2 treatment. Besides, the plant is ubiquitous and the raw material of its aerial part is available for drug manufacturing at respectively low cost, so such drugs are affordable even for the developing countries.

At the same time, the effects of co-administration of *A. podagraria* with widely used antihyperglycemic drugs, such as metformin, have not been verified. It is expedient to investigate combination of metformin with the tincture of *A. podagraria* aerial part because just this preparation has a significant influence on glucose metabolism both in alloxan-induced animals and on the model similar to metabolic syndrome [13, 14], and it seems reasonable to use experimental model that reproduce the pathogenetic mechanisms of the human metabolic syndrome and type 2 diabetes [17, 18].

Therefore, the objective of this study is to determine the influence of *A. podagraria* tincture and its combination with metformin on glucose and lipid metabolism in dexamethasone-treated rats.

The dose of dexamethasone 5 mg/kg subcutaneously was used in the study based on data demonstrating that it leads to severe disorders of glucose metabolism in the adult rat [19], at a close dose of 4 mg/kg significant disorders of lipid metabolism are also seen [20]. As the study was aimed at exploring the possibility of increasing the efficacy of metformin, this drug was used at respectively low dose of 50 mg/kg. Still this dose was found to be effective on the models of glucose metabolism disorders (including insulin resistance) in rats [21] and even on the models of type 1 diabetes [7, 8]. *A. podagraria* tincture was administered at a dose of 1 ml/kg intragastrically, since this dose produces hypoglycemic effect in rats on the model of metabolic disorders that mimic the human metabolic syndrome [14] but does not change glycemia in intact animals [15].

## Methods

### Plant material

The aerial parts of *A. podagraria L.* were collected from natural population in Kharkiv region (Ukraine) in June. Voucher specimens of the species were identified by Ass. Prof. Dr. S.I. Stepanova and deposited at the department of nutriciology and pharmaceutical bromatology (National University of Pharmacy, Kharkiv, Ukraine). The herbal raw material was dried at room temperature and powdered using a standard grinding mill. Then the powder was used for the obtaining of the tincture by double extraction with 70 % ethyl alcohol. The plant material and solvent were taken in 1:5 ratio, the solvent volume was increased according to the swelling index. The solvent was divided into two parts. The plant material was macerated in 2/3 solvent at room temperature for five days accompanying occasional shaking and stirring. The mixture was filtered under vacuum conditions and maceration process was repeated under the same conditions with the rest of the solvent. The obtained liquids were combined into one, kept for two days at 4 °C, filtered and brought to the calculated volume with the solvent. Goutweed tincture is dark green liquid with a characteristic odour. The technology is standard and corresponds to the requirements of State Pharmacopoeia of Ukraine, and was previously described. For the routine standardization of the obtained samples, hydroxycinnamic acids total content was measured in the tincture using UV spectrophotometry. This value was within the range of 0.26–0.36 % [11, 15].

### Drugs and chemicals

Dexamethasone solution for injection (4 mg/ml, KRKA, d. d., Slovenia) and metformin (Sigma-Aldrich Corp., USA) were used in the study. Before administration metformin was dissolved in distilled water and alcohol was removed from *A. podagraria* tincture (ex tempore). Insulin of short action (Actrapid, Novo Nordisk® A/S, 100 U/ml) was used for insulin tolerance test. Commercially-available kits from Filisit-Diagnostika (Ukraine) were used for biochemical assays. Other chemicals used were of analytical grade.

### Animals

Noninbred albino rats breeded in the Central Scientific-Research Laboratory of National University of Pharmacy (Ukraine) were used. Male rats with 180 to 240 g body weight were housed in a well-ventilated animal room at a controlled temperature and relative humidity, on a natural light–dark cycle. Food and water were supplied ad libitum. All the experimental protocols were approved the Bioethics Commission of the National University of Pharmacy (Kharkiv, Ukraine) and were in accordance with “Directive 2010/63/EU of the European Parliament and of the Council of 22 September 2010 on the protection of animals used for scientific purposes”.

### General procedures

After one week of acclimation, the rats were randomly divided into five groups, as follows:

- Group I: intact control (saline solution subcutaneously + tap water intragastrically, *n* = 7);
- Group II: dexamethasone, 5 mg/kg subcutaneously + tap water intragastrically (*n* = 6);
- Group III: dexamethasone, 5 mg/kg subcutaneously + metformin, 50 mg/kg intragastrically (*n* = 6);
- Group IV: dexamethasone, 5 mg/kg subcutaneously + *A. podagraria* tincture, 1 ml/kg intragastrically (*n* = 6);
- Group V: dexamethasone, 5 mg/kg subcutaneously + metformin, 50 mg/kg intragastrically + *A. podagraria* tincture, 1 ml/kg intragastrically (*n* = 6).

The interval between the administration of GW tincture and metformin equalled 40 min to minimize the interaction at the level of absorption. The drugs were administered once a day, between the 10 am and 12 pm. Animals were fasted for 12 h before the tests and taking final blood samples but they were allowed free access to tap water.

The general duration of the experiment was 5 days. Insulin tolerance test was performed on day 3, oral glucose tolerance test – on day 4. On day 5 of the experiment (40 min after the drugs administration) heparinized blood samples were drawn by exsanguination from barbiturate-anesthetized animals. Plasma (the anticoagulant heparin in vitro) was separated immediately by centrifugation. Liver tissue samples were collected for glycogen level measurement.

### Insulin tolerance test

40 min after administration of the drugs, insulin was injected at a dose of 1.0 U/kg. Blood samples for glucose determination were obtained from a cut at the tip tail at 0 and 30 min [18].

### Oral glucose tolerance test

40 min after administration of the drugs 30 % glucose solution was given intragastrically at a dose of 3.0 g/kg. Blood samples for glucose determination were obtained from a cut at the tip tail at 0, 30, 60, and 120 min [18]. The total area under the blood glucose curve was calculated using the trapezoidal method, the average glycemia value was also determined.

### Biochemical analysis

Glucose concentration in all samples was measured using the glucose oxidase method [22].

Liver glycogen was isolated through precipitation with ethanol after alkaline hydrolysis of the liver samples and determined as glucose after acid hydrolysis and subsequent neutralization [23].

Triglycerides concentration and total cholesterol content in plasma were determined using enzymatic methods [24, 25], HDL cholesterol (HDL-C) – using phosphotungstate-Mg^2+^ precipitation and enzymatic cholesterol assay [26], plasma total lipids level – by the reaction with phospho-vanillin reagent [27].

Atherogenic index was calculated by using the following formula [28]:

$$ \mathrm{atherogenic}\;\mathrm{index}=\left(\mathrm{total}\;\mathrm{cholesterol} - \mathrm{H}\mathrm{D}\mathrm{L}-\mathrm{C}\right)/\mathrm{H}\mathrm{D}\mathrm{L}-\mathrm{C} $$

The formula of Friedewald et al. (LDL-C = total cholesterol – HDL-C – (triacylglycerols/2.2)) was used to determine LDL cholesterol (LDL-C) as it has been shown that it is correct in rats whose HDL-C constitutes 75 % (or more) of total serum (plasma) cholesterol [29], and our data approached this value.

The adequacy of heparinized plasma use in the study of the lipid metabolism values is substantiated by the data about low basal level and activity of lipoprotein lipase in rat plasma [30], and the absence of enzymatic activity of the monomeric form of lipoprotein lipase previously released from endothelium [31].

### Statistical analysis

Medians, 25 % and 75 % percentiles (upper and lower quartiles) were calculated as recommended for biomedical research [32]. The traditionally used arithmetic means and their standard errors (M ± m) are also given. The comparison of the central tendencies of independent samples was performed by the criterion of Mann–Whitney U (taking into account a problematical character of multiple comparisons in pharmacology and toxicology [33]). To determine the relationship between the individual parameters, the Spearman’s correlation coefficient of ρ was used.

## Results and discussion

Dexamethasone-induced model is considered a suitable model for the investigation of drugs influencing the pathogenetic mechanisms of type 2 diabetes and metabolic syndrome. Diabetogenic effects are realized through dysregulation of glucose homeostasis in liver, muscles, adipose tissue. Reduction in peripheral insulin sensitivity [34] may be compensated by the intensified pancreatic β-cells function, but, as the latter is also directly affected by glucocorticoids, over time decompensation arises. In addition to the peripheral action, steroids modulate the synthesis and release of hormones associated with the development of diabetes, namely glucagon, somatostatin, amylin, ghrelin, leptin [17, 18].

**Fig. 1:**
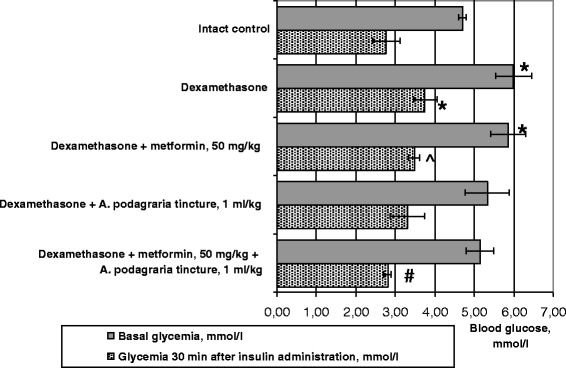
Influence of *A. podagraria* tincture and metformin on insulin sensitivity in rats receiving dexamethasone. Values are expressed as Mean ± S.E.M; * – *p* < 0.05 compared to intact control; # – *p* < 0.02 compared to dexamethasone (untreated); ^ – *p* < 0.01 compared to the group receiving metformin and *A. podagraria* tincture

We observed a significant increase in basal glycemia induced by dexamethasone on day 3 of the experiment (Fig. 1), such change was also evident on day 4, but did not reach statistical significance because of inter-individual differences (Table 1). The possible reason for the fasting hyperglycemia is the well known ability of glucocorticoids (at high doses) to promote gluconeogenesis in hepatic tissue [17]. Despite of similar percent change from baseline in the short insulin test (medians of this value equalled 40–46 % in all groups), blood glucose level after insulin injection was significantly higher in animals treated with dexamethasone than in intact rats (Table 1) indicating inability to utilize blood glucose even under the influence of insulin.

Metformin per se did not normalize basal level of glycemia (Fig. 1, Table 1) but this value tended to return to the normal state in animals receiving GW tincture per se or with metformin on day 4, and on day 4 there were statistically significant differences in basal glycemia between groups of rats receiving metformin and metformin combined with GW tincture. 30 min after insulin injection, the lowest glycemia against a background of dexamethasone was registered in animals treated with GW tincture and metformin combination (*p* < 0.02 when compared with dexamethasone treatment only, Fig. 1). The involvement of GW substances into the hypoglycemic activity of the combination in the short insulin test is confirmed by the statistically significant difference in glycemia between groups of rats receiving metformin per se and in combination with GW tincture. As basal glycemia on day 3 showed a clear tendency to reduction, the percent change from baseline in animals treated with the investigated combination did not changed noticeably. The GW tincture per se shown a decreasing trend on glycemia (both basal and after insulin injection) which approximated to the values of rats receiving metformin and did not differ significantly from intact control values (Fig. 1).

**Table 1:** Influence of *A. podagraria* tincture and metformin on the results of oral glucose tolerance test in rats receiving dexamethasone; Mean ± S.E.M; Q_50_ (Q_25_–Q_75_), *n* = 5–6 in each group

Groups	Plasma glucose during oral glucose tolerance test, mmol/l				AUC, mmol × min/l	Average glycemia value, mmol/l
	Basal level	30 min	60 min	120 min		
Intact control	4.38 ± 0.54 **4.91** (3.42–5.34)	5.08 ± 0.42 **5.34** (4.48–5.83)	6.37 ± 0.77 **6.60** (5.60–6.83)	3.91 ± 0.35 **3.84** (3.54–4.06)	622 ± 52.8 **682** (551–707)	4.93 ± 0.39 **5.34** (4.56–5.44)
Dexamethasone	6.01 ± 0.56 **5.44** (4.98–6.68)	7.56 ± 0.72^***^ **6.60** (6.58–7.86)	7.23 ± 0.54 **6.60** (6.25–8.15)	6.22 ± 0.43*** **5.87** (5.55–6.63)	829 ± 54.9* **785** (720–935)	6.75 ± 0.44** **6.49** (5.83–7.77)
Dexamethasone + metformin, 50 mg/kg	6.27 ± 0.53 **6.68** (5.18–7.14)*****	6.33 ± 0.73 **6.58** (5.27–7.04)	6.09 ± 0.76 **6.25** (5.20–6.70)	4.93 ± 0.33**** **4.91** (4.91–5.11)	701 ± 69.1 **708** (650–742)	5.83 ± 0.53 **5.69** (5.64–6.14)
Dexamethasone + *A. podagraria* tincture, 1 ml/kg	5.31 ± 0.58 **4.78** (4.31–5.81)	8.20 ± 1.46* **6.49** (5.92–9.49)	7.18 ± 1.27 **6.00** (5.13–8.25)	6.07 ± 1.05 **6.24** (4.79–7.95)	831 ± 136 **724** (611–980)	6.69 ± 1.05 **5.88** (5.00–7.85)
Dexamethasone + metformin, 50 mg/kg + *A. podagraria* tincture, 1 ml/kg	4.24 ± 0.62 **4.35** (3.26–5.17)	6.68 ± 0.74* **7.53** (6.02–7.68)	5.26 ± 0.72**** **6.03** (3.66–6.40)	4.94 ± 0.78 **5.11** (4.27–6.29)	649 ± 62.1 **638** (576–757)	5.28 ± 0.45**** **5.12** (5.11–5.82)

* – *p* < 0.05 compared to intact control; ** – *p* < 0.02 compared to intact control; *** – *p* < 0.01 compared to intact control

**** – *p* < 0.05 compared to dexamethasone (untreated); ***** – *p* < 0.05 compared to the group receiving metformin and *A. podagraria* tincture

Medians are highlighted in bold

In the oral glucose tolerance test, in addition to the changes in basal glycemia described above, a significant increment of glycemia 30 min after glucose load was seen in dexamethasone-treated rats (Table 1). The increase in early postprandial glycemia due to the loss of early insulin release is considered to play an essential role in type 2 diabetes [35]. Besides, the changes in glucose absorption are possible. According to the data [36], the rate of appearance in plasma of gut-derived glucose increased twice in rats treated with dexamethasone (even at low doses). At the same time, the incompleteness of glucose utilization by the tissues is reflected on significantly increased glycemia 120 min after its intragastrical administration (while in intact animals glycemia at this time was reduced to the level lower than the basal one). The mentioned changes led to a marked increment of the area under the blood glucose curve as well as average glycemia value.

The significant hypoglycemic effect of metformin was seen only after 120 min. GW tincture per se (as well as metformin per se) tended to normalize average glycemia value but the effect was not statistically significant. The lowest glycemia at baseline and 60 min after glucose load was seen in animals receiving combined treatment and the latter (as well as average glycemia value) had statistically significant differences with the group receiving dexamethasone. The area under the blood glucose curve in this group approximated to the value of intact rats (Table 1).

The insufficiency of metformin hypoglycemic action may be explained by the respectively low dose used. Thus, amelioration of dexamethasone-induced hyperglycemia and insulin resistance in part by increasing glucose disposal into skeletal muscle was proven for metformin at significantly higher dose (250 mg/kg) [37].

Our data described above show that the possibility to increase metformin activity (possibly improving the safety profile and reducing the costs) by combining it with GW tincture could be practically realized. The tincture at the dose used does not cause significant glycemia decrease (Table 1), still it is able to enhance the effects of metformin as basal glycemia is lowered when metformin is given together with GW tincture (with statistically significant differences between the groups receiving metformin per se and in combination with GW tincture).

Hyperglycemic influence of dexamethasone was also evident on day 5 under conditions of anaesthesia (Table 2). The values of rats treated with metformin, GW tincture or their combination had no statistically significant differences compared with the data of intact control as well as data animals receiving dexamethasone (Table 2).

**Table 2:** Influence of *A. podagraria* tincture and metformin on glucose metabolism in rats receiving dexamethasone; Mean ± S.E.M; **Q**_**50**_ (Q_25_–Q_75_), *n* = 5–7 in each group

Groups	Liver glycogen, mg/g	Glucose level in plasma after anaesthesia, mmol/l	Coefficients of correlation between liver glycogen and plasma glucose
Intact control	13.7 ± 2.72 **13.8** (9.44–17.1)	8.13 ± 0.42 **8.76** (7.62–8.83)	**+0,61** NS
Dexamethasone	39.0 ± 2.76*** **37.7** (34.1–40.8)	9.49 ± 0.46** **9.03** (8.90–9.37)	**−0,37** NS
Dexamethasone + metformin, 50 mg/kg	33.3 ± 5.31*** **28.0** (24.4–38.3)	8.98 ± 1.14 **8.69** (7.30–10.7)	**+0,70** NS
Dexamethasone + *A. podagraria* tincture, 1 ml/kg	36.0 ± 3.13*** **36.0** (34.3–40.2)	8.44 ± 0.49 **8.80** (7.60–9.33)	**+0,30** NS
Dexamethasone + metformin, 50 mg/kg + *A. podagraria* tincture, 1 ml/kg	32.7 ± 3.36*** **34.0** (29.1–37.8)	8.60 ± 0.31 **8.33** (8.13–9.21)	**+0,77** NS

** – *p* < 0.02 compared to intact control; *** – *p* < 0.01 compared to intact control

Medians are highlighted in bold

Glycogen content in the liver of dexamethasone-treated animals increased dramatically – in 184 %. Despite the possibility of glucocorticoids influence on the insulin signalling cascade in the liver, leading to the impairment of the hepatic glycogen synthesis [17] and controversial data obtained in vitro (dose- and time-dependent, biphasic effect on hepatocytes glycogen metabolism) [38], in conscious animals dexamethasone is known to stimulate liver glycogen deposition and hepatic glucose synthesis and, as suggested by S. Baque et al. (1996) [39], glucocorticoids may contribute to both glycogenolysis and gluconeogenesis while sustaining increased liver glucose output. Positive correlation between the level of liver glycogen and plasma glucose registered in the group of intact animals disappeared in dexamethasone-treated rats (Table 2). GW tincture was not able to influence the liver glycogen level. Metformin is usually claimed to stimulate glycogenesis, still there is evidence that it inhibits basal glycogen synthesis and cellular glycogen contents [40] and in our study this drug tended toward the reduction of liver glycogen (per se and in combination with the investigated tincture), and the correlation coefficient between the level of liver glycogen and plasma glucose approximated to the intact group value (Table 2). The regulatory mode of action may be assumed for metformin and the absence of the statistically significant effect may be explained by the low dose used.

**Fig. 2:**
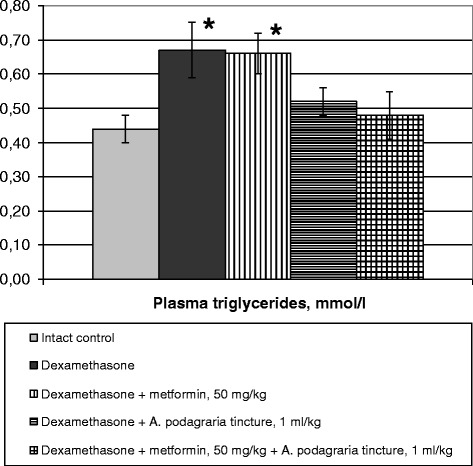
Influence of *A. podagraria* tincture and metformin on plasma triglycerides level in rats receiving dexamethasone. Values are expressed as Mean ± S.E.M; * – *p* < 0.05 compared to intact control

As to the lipid metabolism changes, dexamethasone significantly increased plasma triglycerides and tended to raise plasma total lipids level (Fig. 2, Table 3). The increment of total cholesterol content was not statistically significant (Table 3) and was caused by the increase in HDL and VLDL cholesterol without changes in LDL cholesterol concentration and atherogenic index (Table 3). Similar data are available in the literature: dexamethasone at a dose of 5 mg/kg perorally during 7 days influenced only on VLDL lipoprotein level with non-significant increase in total plasma cholesterol level in rats [41]. This is consistent with the known ability of glucocorticoids to activate the expression of several genes encoding enzymes in triglycerides synthesis and cause lipids redistribution with the lipolysis in adipocytes as well as triglycerides accumulation in the liver [42].

**Table 3:** Influence of *A. podagraria* tincture and metformin on plasma values of lipid metabolism in rats receiving dexamethasone; Mean ± S.E.M; Q_50_ (Q_25_–Q_75_), *n* = 5–6 in each group

	Total cholesterol, mmol/l	HDL cholesterol, mmol/l	LDL cholesterol, mmol/l	VLDL cholesterol, mmol/l	Atherogenic index	Total lipids, g/l
Intact control	2.38 ± 0.20 **2.36** (2.20–2.51)	1.72 ± 0.13 **1.64** (1.47–1.93)	0.62 ± 0.05 **0.58** (0.57–0.66)	0.20 ± 0.02 **0.20** (0.17–0.25)	0.39 ± 0.07 **0.44** (0.26–0.50)	2.48 ± 0.18 **2.56** (2.10–2.70)
Dexamethasone	2.71 ± 0.30 **2.64** (2.20–3.02)	2.03 ± 0.22 **2.16** (1.73–2.20)	0.55 ± 0.08 **0.59** (0.47–0.59)	0.30 ± 0.04* **0.32** (0.24–0.36)	0.42 ± 0.04 **0.42** (0.38–0.45)	2.82 ± 0.23 **2.88** (2.61–2.94)
Dexamethasone + metformin, 50 mg/kg	2.62 ± 0.19 **2.49** (2.32–2.82)	2.17 ± 0.14 **2.20** (1.89–2.29)	0.31 ± 0.10 **0.27** (0.23–0.35)	0.30 ± 0.04* **0.28** (0.25–0.34)	0.21 ± 0.06**** **0.23** (0.10–0.25)	2.60 ± 0.26 **2.54** (2.13–2.77)
Dexamethasone + *A. podagraria* tincture, 1 ml/kg	2.80 ± 0.15* **2.76** (2.53–2.84)	2.31 ± 0.18** **2.11** (2.07–2.36)	0.24 ± 0.05***,****, ******* **0.28** (0.18–0.33)	0.24 ± 0.02 **0.26** (0.23–0.28)	0.22 ± 0.03*****,****** **0.23** (0.21–0.25)	2.91 ± 0.29 **2.79** (2.41–3.16)
Dexamethasone + metformin. 50 mg/kg + *A. podagraria* tincture, 1 ml/kg	2.92 ± 0.11* **2.87** (2.78–2.92)	2.19 ± 0.06* **2.12** (2.09–2.23)	0.51 ± 0.08 **0.43** (0.38–0.66)	0.22 ± 0.03 **0.20** (0.16–0.25)	0.33 ± 0.03**** **0.33** (0.28–0.38)	2.61 ± 0.17 **2.51** (2.35–2.83)

* – *p* < 0.05 compared to intact control; ** – *p* < 0.02 compared to intact control; *** – *p* < 0.01 compared to intact control

**** – *p* < 0.05 compared to dexamethasone (untreated); ***** – *p* < 0.01 compared to dexamethasone (untreated)

****** – *p* < 0.05 compared to the group receiving metformin and *A. podagraria* tincture

******* – *p* < 0.02 compared to the group receiving metformin and *A. podagraria* tincture

Medians are highlighted in bold

Under the influence of GW tincture, but not metformin, concentration of plasma triglycerides had no statistically significant differences compared with the intact control value (Fig. 2). Metformin and its combination with the investigated herbal drug also tended towards the normalization of the total lipids level (Table 3). The tincture per se or with metformin caused a significant increase in HDL cholesterol together with total plasma cholesterol level (Table 3). Unexpectedly, the ability of GW tincture to reduce LDL cholesterol content and the same tendency seen against a background of metformin were eliminated when these preparations were administered together, and the significant differences in atherogenic indices were seen between the groups receiving the tincture and its combination with metformin. Spearman’s coefficient of correlation between plasma total cholesterol and HDL cholesterol was not statistically significant in this group (0.26, *p* > 0.05), while in all other groups was within the range of 0.83–0.98 (*p* < 0.05 in all cases).

Hypolipidemic properties of metformin are widely known, still it has been shown that, in type 2 diabetes mellitus, metformin does not affect plasma HDL cholesterol and plasma triglycerides directly (more than might be expected from its glucose-lowering effect) [43]. This drug action is considered to be dose-dependent and the absence of its significant influence at low dose is the expected result.

The question about the active components and mechanisms of action of GW tincture that contribute to the increase of metformin efficacy logically arises. Among the substances present in the tincture, hydroxycinnamic acids are the most possible active compounds [10–12]. Macroelements, especially potassium and magnesium, may mediate favourable metabolic activity, still their effect is hardly the principal one, concerning the relatively short course of treatment used in our study and lower level of these compounds extractable with ethanol in comparison with water [12]. Protein-polysaccharide complex is beleived to contribute to the activity of GW extract but it is not present in sufficient quantity in the tincture [11, 12].

A great body of evidence exists about the efficacy of hydroxycinnamic acids and flavonoids in metabolic syndrome and diabetes [5, 6, 44]. Chlorogenic acid has been recently claimed to modulate glucose and lipid metabolism in vivo and to mediate the favourable effects of the herbal extracts with well-known antidiabetic activity, such as Morus alba leaf extract [45] as well as coffee [5]. In our study the clear tendency towards decrease in basal glycemia was seen in animals receiving the investigated tincture, and it may be linked to the inhibitory effect of chlorogenic acid on gluconeogenesis (through the influence on glucose-6-phosphate translocase that is verified in vitro and in diabetic animals) [46]. As the content of hydroxycinnamic acids (in terms of chlorogenic) in *A. podagraria* tincture reaches 0.36 %, the animals received 3.6 mg/kg of this compound with the tincture at a dose of 1 ml/kg. This dose is close to the dose of caffeic acid 5 mg/kg investigated by K. Karthikesan et al. [47], but somewhat lower than the doses of chlorogenic acid used in the studies in vivo [5, 45], that corresponds to the absence of the statistically significant hypoglycemic effect of the tincture at the dose used.

As to the lipid metabolism influence, chlorogenic acid is even characterized as “an anti-obesity natural molecule” [48], it is known to reduce hepatic triglycerides level in obese mice (at respectively high dose of 30–60 mg/kg) [5], and GW tincture exerts such effect in rats receiving ethanol (but at higher dose of 5 ml/kg) [49]. It has been shown recently that chlorogenic acid is one of the active ingredients in the crude herbal drug counteracting chronic ethanol-induced hepatic lipid accumulation through MAPK/SREBP-1c-dependent and -independent signalling pathways [50] and in vitro it increases the efflux of total cholesterol and triacylglycerols to the hepatocytes, inhibits HMG-CoA reductase activity [51]. In addition to chlorogenic acid, other hydroxycinnamic acids may mediate pharmacological activity of GW drugs. Furthermore, as the dose of the tincture containing respectively low dose of chlorogenic acid was found to increase the effects of metformin, the other substances, most probably flavonoids and other phenolic compounds are involved into pharmacological activity of the former. It is beleived that the favourable metabolic effects of coffee are the result of the synergistic polyphenols action, and enhancement of caffeine and chlorogenic acid effects in combined administration was proven experimentally in the study of G. Zheng et al. (2014) that addressed hepatic lipid metabolism in mice [52]. The limitations in such studies of crude herbal drugs and problems in preclinical data extrapolation into humans were actively discussed [48]. Nevertheless, the synergistic mechanisms of the herbal drugs constituents are generally recognized [53].

Besides hydroxycinnamic acids, flavonoids quercetin, kaempferol, and their derivatives are among active substances of GW tincture [11, 12]. Health benefits of the dietary flavonoids, including management of metabolic syndrome, obesity, and diabetes mellitus are widely known [6]. As dexamethasone downregulates PI3K in rodent skeletal muscle cells and suppresses insulin-induced translocation of GLUT4 in myotubes [17], it may be reasonable to mention that kaempferol increases glucose uptake and quercetin stimulates GLUT4 translocation and expression in skeletal muscles [6]. Furthermore, the antidiabetic potential of flavonoids has recently been associated mainly with the modulatory effects on glucose transporters including promotion of GLUT-4 translocation [44]. Flavonoids are able to increase the expression of LDL-receptors in the liver (quercetin) [54], suppress mRNA expression and activity of the enzymes involved in fatty acids and triglycerides biosynthesis in the liver (quercetin, rutin) [55], modulate lipid metabolism through SREBP-1c, PPAR-α, and PPAR-γ regulation and increased expression of acyl-CoA oxidase (kaempferol) [56]. Also flavonoids are able to reduce the level of the mediators linked to inflammation and oxidative stress in the adipose tissue [6].

So there are enough data confirming the normoglycemic and hypolipidemic effects of GW active components. It is much more difficult to explain the disappearance of the influence on plasma lipoproteins ratio after combined administration of metformin and the tincture. The results of this study do not allow elucidating the mechanism of this interaction and further experiments are required. Unfavourable pharmacokinetic interactions between metformin and GW components were not likely to happen because hypoglycemic activity was present and even enhanced after combined administration of these preparations. As described above, time interval between these drugs administration allowed minimizing interactions at the level of absorbtion, and interactions at the level of metabolism are not typical for metformin. Considering pharmacodynamical interactions, it may be assumed that the different targets exist for metformin and GW components and, taking into account the complexity of the metabolic regulatory systems, no desired effect on lipoproteins spectrum is seen if these targets are activated simultaneously. In the present article we have not addressed these mechanisms, but indirect evidence can be found in the literature. It has been shown in vitro that MPK activators including metformin inhibit transcriptional activities of PPAR-α and PPAR-γ [57] and activation of PPAR-α expression that facilitates lipid clearance in the liver is considered to mediate the efficacy of such GW active components as kaempferol, chlorogenic and neo-chlorogenic acid in high-fat diet-fed animals [56, 58, 59].

In general, the specific mechanisms of herbal drugs interactions with conventional drugs are not studied enough. Most of the data available are limited to the evidence of the enhancement of metformin antihyperglycemic action by herbal extracts [7, 8]. In-depth study of GW preparations interaction with metformin (especially in the context of normoglycemic effect) also should be addressed in future. Still there are data in the literature that partially support our results: chlorogenic acid as well as ferulic acid show a synergistic effect with metformin on the glucose uptake by myotubes. Chlorogenic acid increases GLUT4 expression via PI3K independent pathway, whereas ferulic acid activity is realized through PI3K dependent pathway [9]. Such effects of of GW components may determine its permissive effect om the action of metformin that is becoming possible at lower doses.

## Conclusions

In conclusion, for the first time, GW tincture combined with the respectively low dose of metformin increased the effect on the latter on the basal glycemia in dexamethasone-treated rats. It also showed a permissive effect on the action of metformin in the short insulin test indicating the improvement in the peripheral insulin sensitivity. The efficacy of the investigated combination was partially registered in the oral glucose tolerance test (the lowest area under glucose curve and average glycemia value were seen in this group). Dexamethasone-induced dyslipidemia was partially corrected by the investigated preparations: although the statistically significant reduction in LDL cholesterol content was eliminated by simultaneous metformin administration, the decrease in triglycerides level and increment of HDL cholesterol content (caused by the tincture), the tendency towards the decrease in total lipids level (resulting from metformin administration) were observed against a background of the investigated combination. In-depth study of GW preparations interaction with metformin in the treatment of glucose metabolism disorders is expedient.

## Abbreviations

GW, goutweed; HDL, high-density lipoproteins; HDL-C, high-density lipoproteins cholesterol; LDL, low-density lipoproteins; LDL-C, low-density lipoproteins cholesterol
